# Scanned carbon beam irradiation of moving films: comparison of measured and calculated response

**DOI:** 10.1186/1748-717X-7-55

**Published:** 2012-04-02

**Authors:** Christoph Bert, Daniel Richter, Marco Durante, Eike Rietzel

**Affiliations:** 1 GSI Helmholtzzentrum für Schwerionenforschung, Planckstr. 1, 64291 Darmstadt, Germany; 2 TU Darmstadt, Institut für Festkörperphysik, Hochschulstr. 6, 64289 Darmstadt, Germany; 3 Siemens AG, Healthcare Sector, Imaging & Therapy, Particle Therapy, Hofmannstr. 26, 91052 Erlangen, Germany

## Abstract

**Background:**

Treatment of moving target volumes with scanned particle beams benefits from treatment planning that includes the time domain (4D). Part of 4D treatment planning is calculation of the expected result. These calculation codes should be verified against suitable measurements. We performed simulations and measurements to validate calculation of the film response in the presence of target motion.

**Methods:**

All calculations were performed with GSI's treatment planning system *TRiP*. Interplay patterns between scanned particle beams and moving film detectors are very sensitive to slight deviations of the assumed motion parameters and therefore ideally suited to validate 4D calculations. In total, 14 film motion parameter combinations with lateral motion amplitudes of 8, 15, and 20 mm and 4 combinations for lateral motion including range changes were used. Experimental and calculated film responses were compared by relative difference, mean deviation in two regions-of-interest, as well as line profiles.

**Results:**

Irradiations of stationary films resulted in a mean relative difference of -1.52% ± 2.06% of measured and calculated responses. In comparison to this reference result, measurements with translational film motion resulted in a mean difference of -0.92% ± 1.30%. In case of irradiations incorporating range changes with a stack of 5 films as detector the deviations increased to -6.4 ± 2.6% (-10.3 ± 9.0% if film in distal fall-off is included) in comparison to -3.6% ± 2.5% (-13.5% ± 19.9% including the distal film) for the stationary irradiation. Furthermore, the comparison of line profiles of 4D calculations and experimental data showed only slight deviations at the borders of the irradiated area. The comparisons of pure lateral motion were used to determine the number of motion states that are required for 4D calculations depending on the motion amplitude. 6 motion states per 10 mm motion amplitude are sufficient to calculate the film response in the presence of motion.

**Conclusions:**

By comparison to experimental data, the 4D extension of GSI's treatment planning system TRiP has been successfully validated for film response calculations in the presence of target motion within the accuracy limitation given by film-based dosimetry.

## Background

Radiotherapy of tumors influenced by organ motion benefits from dedicated treatment planning. Our treatment planning system for scanned particle beams (*Treatment planning for Particles*, TRiP) [1] was extended to include 4D functionality, i.e. time resolved dose calculation and treatment plan optimization [2]. The purpose of this contribution is to report measurements and corresponding film response calculations which were performed to confirm 4D calculations with TRiP. Such 4D calculations can, e.g., be used in treatment planning for patients with a moving tumor to check the robustness of the optimized 4D treatment plan by multiple calculations with simulated, time resolved, data of tumor motion and beam delivery.

Experiments were performed with a raster-scanned carbon beam [3] at GSI Helmholtz Centre for Heavy Ion Research in Darmstadt, Germany. Dose distributions delivered by scanned pencil beams are conformal to the target volume and allow for homogeneous coverage. But dose distributions are also sensitive to intrafractional organ motion because the combination of beam scanning and target motion represents a double dynamic system and typically results in interplay. Interplay causes deterioration of the dose distribution leading to hot and cold spots [4-6]. With techniques like gating [7], rescanning [4], or beam tracking [8,9], treatment of tumors that are subject to intrafractional organ motion like respiration with scanned particle beams can potentially be improved.

For a specific treatment plan, the interplay pattern of scanned beam and target motion is influenced by the target motion parameters (period, initial phase, amplitude) and the intensity controlled beam scanning process, especially scan speed which primarily depends on the particle extraction from the synchrotron [5]. 4D calculations incorporate these parameters to determine the interplay pattern or mitigated interplay pattern in case of beam tracking, gating, or rescanning. In a clinical setting, multiple calculations with varying motion and extraction parameters and/or mitigation techniques could be performed as part of treatment planning to estimate the range of possible outcomes. It is thus very important, that these calculations are valid. Their validity can be assessed by irradiating without motion mitigation, i.e. by producing interplay patterns. Validity is achieved if the calculated outcome is within the achievable experimental accuracy. We performed experiments with moving radiographic films as detector without using a motion mitigation technique. Radiographic films have a very high lateral spatial resolution and are thus ideally suited to detect interplay patterns. For measurements in a 3D irradiation field, multiple films can be stacked in depth alternated with spacers such as Lucite plates. In such 3D measurements the agreement of measured and calculated film blackening deviates between 10% and 30% due to the mixed radiation field of a carbon beam [10].

In order to allow a detailed study of the potential deviations between measurement and calculation we intentionally limited ourselves to measuring the film response and left-right target motion with depth-modulation by a stationary absorber positioned proximal of the target volume. Non-rigid motion that will be present in patients was not introduced on purpose. We designed the treatment plans in accordance with film (stacks) as target. These planar targets lead to irradiations with (multiple) pristine Bragg-peaks rather than treatment plans with a spread-out Bragg-peak (SOBP). In an SOBP irradiation, mitigation of interplay effects to some extent takes place intrinsically, e.g., due to rescanning effects in proximal target regions [11]. Thus, cause of deviations cannot be precisely studied. Since SOBPs are essential in clinical use, appropriate investigations have been performed by Gemmel et al. using Chinese hamster ovary cells to also incorporate the biological effect of carbon beams [12]. In addition, irradiations to a 3D geometry with an array of ionization chambers as detector are currently ongoing. Apart from comparing measured with calculated results that will validate the calculation we studied the impact of the number of motion states used for 4D calculation on the precision of the calculation in dependence on the motion amplitude.

## Methods

### Experimental methods

#### Setup

**Figure 1:**
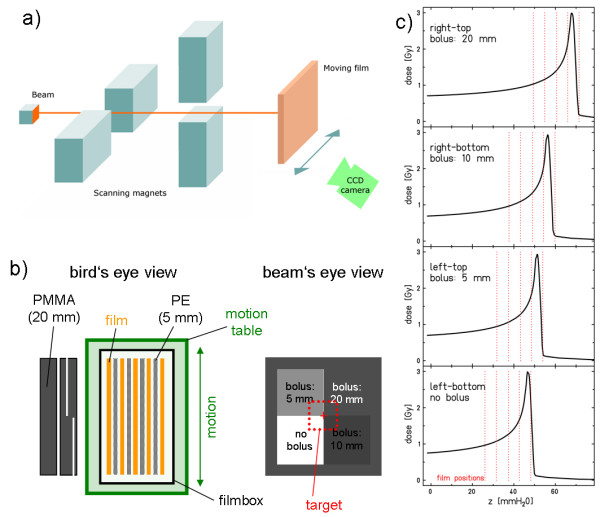
**Schematic drawing of the experimental setup**. The particle beam was accelerated by a synchrotron and scanned over the moving radiographic film with the scanning magnets. **a**) For experiments with translational motion the films were positioned free in air moving left-right in beam's eye view. The motion was recorded in temporal correlation to the dose delivery by a calibrated CCD camera that monitored an infrared LED attached to the sliding table. **b**) In a second series of experiments an absorber (PMMA) with 4 different thicknesses that was positioned proximal to the moving table introduced range changes. As detector a stack of 5 films was used. Their positions relative to the Bragg peak is indicated in **c**).

A schematic drawing of the setup is shown in Figure 1. The irradiations were performed in the patient treatment room at GSI. At isocenter, a periodically moving table was installed. The table moved radiographic films (Kodak X-Omat V, Kodak GmbH, Stuttgart) as detectors in left-right direction in beam's eye view. Motion amplitude, period, and initial motion states were controlled and recorded. In a first set of experiments, the effects of lateral target motion were investigated: Films were positioned free in air, no absorber material was used (Figure 1a). A second series of experiments was performed to assess range effects in the presence of target motion. An absorber with four different thicknesses was positioned stationary proximal to the motion table (Figure 1b). A stack of five films between absorber materials was used for these measurements (Figure 1b, c).

To measure the motion of the moving table, a calibrated CCD camera monitored the movement of an IR-LED attached to the table. Data were acquired on a PC running LabVIEW (National Instruments Germany GmbH, LabVIEW, Version 7.1, Munich, Germany) with the Image Vision package (National Instruments Germany GmbH, Image Vision, Munich, Germany). The beam extraction pattern of the synchrotron was determined temporally correlated by measuring the irradiation time of each beam position during raster scanning.

#### Treatment plan and treatment delivery

Different treatment plans were used for the two experimental setups. For left-right motion without range modulation, a single iso-energy slice was used (carbon beam, 272 MeV/u). A quadratic area of 11 × 11 cm^2 ^(50% iso-dose) was irradiated to ~0.9 Gy, raster points (7.7 mm FWHM pencil beams) were placed on a regular 2 mm grid (3013 beam positions), and scanned intensity-controlled [3] line-by-line with the faster scanning direction horizontally (x-axis, parallel to target motion). For the second setup including range changes, the treatment plan was based on a CT scan of the absorber and optimized such that the beam stops before the 5th film in the stack (see Figure 1c) for each absorber thickness. This setup results into four main beam-energies of 148, 155, 163, and 181 MeV/u for the four segments of different absorber thickness of 0, 5, 10, and 20 mm PMMA, respectively. The plan was optimized on a 2 mm grid (2179 beam positions), at 6.5 mm FWHM beam spot size to a lateral area of 72 × 72 mm^2 ^(50% iso-dose).

Target motion was started in correlation with the irradiation. Before delivery of the dose pattern to a moving film, two cross-shaped or quadratic patterns were irradiated on the static film to define a precise 2D coordinate system. By means of the coordinate system the results of the experiments can be related to the results of the calculation.

### Calculation of the expected film response

Calculation of the expected dose distribution in 4D, i.e. in the presence of organ motion was introduced shortly after 4DCT acquisitions became available [13,14]. The initial work focused on 3D conformal photon beam therapy and proton beam delivery with a passively shaped beam. For 4D calculation for scanned carbon ion beam therapy, TRiP was extended by Bert & Rietzel [2]. The basic principles of TRiP4D follow the ideas of Rietzel et al. and Keall et al. and are now also used to calculate the film response in the presence of motion. A brief summary of the underlying strategy follows.

Dose and film response calculations are based on:

i) the parameters of the treatment plan (beam position, beam energy, beam width, particles per beam position) and especially the scanning order of the beam positions,

ii) amplitude based motion states describing the time dependent target positions,

iii) translation vectors from all motion states to a reference motion state,

iv) the measured target motion data, and

v) scanning progress given by the measured irradiation time of each raster point.

The underlying principle is similar to 4DCT data acquisition: Based on (iv) target motion data and (v) scanning progress each rasterpoint of the delivered treatment plan (i) can be attributed to one of the motion states (ii). The motion amplitude detected with the CCD camera was used to determine the corresponding motion state. The peak-to-peak amplitude was divided into equally spaced motion states that correlate to specific positions of the target volume. Corresponding to each motion state, a sub-treatment plan containing the subset of raster points delivered during this motion state was constructed. Sub-treatment plans are used in combination with the corresponding motion state as quasi-static treatment plans and result in quasi-static sub-dose distributions (or sub-film responses). Before summation of all sub-dose distributions to derive the overall dose distribution, each of them is transformed to a reference motion state using the translation vectors (iii).

For calculation of film responses, the non-linear dose-response [15] of films has to be considered. The background-corrected relative blackening *S/S_0 _*of radiographic films can be described by

(1) $$\frac{S}{S_{0}}=1-\exp\left(-mD\right)$$

where *S*_0 _is the experimentally determined saturation level, *m *the slope of the blackening curve, and *D *the used dose-level. The summed relative blackening of the film *S*_tot_/*S*_0 _is not the sum of the transformed sub-film responses *S*_i_/*S*_0 _because the non-linear response *S*_tot_/*S*_0 _is determined as:

(2) $$\frac{S_{\text{tot}}}{S_{0}}=1-\exp\left(-\sum_{1}^{N_{CT}}{\left(mD\right)}_{i}\right)$$

where *N*_CT _is the number of motion states and thus sub-film response distributions. The saturation level *S*_0 _is determined experimentally according to Spielberger et al. [10]. The slope *m *is particle and energy dependent and belongs to the base data which are calculated based on the slope of a ^60^Co blackening curve [16]. For these calculations, analysis of the 3D measurement data reported by Spielberger et al. results in an expected accuracy of 10-30% when comparing to measured data [10]. For the experiments with the film stack (Figure 1b), calculated data are plotted with an error bar of 10% to address the accuracy of the modeled film responses in a mixed radiation field.

For the experiments without absorbers (Figure 1a) fragmentation and energy degradation are negligible and *m *can be measured for the energy of the primary carbon beam. For this measurement, a single film is irradiated with several independent areas (e.g., multiple squares of 5 × 5 cm^2^) of different dose levels. For each area, *S *is determined with a densitometer and corrected by the background measured in unirradiated parts of the film. Using equation 1, *m *and *S_0 _*as well as their errors Δ*m *and Δ*S_0 _*are then determined by a fitting algorithm. We determined the error of the calculated relative film response *S*_tot_/*S*_0 _(equation 2) by error propagation and show it as error bars on line profiles.

To transform the sub-dose distributions of the different motion states to the reference motion state, a one-dimensional translation vector was sufficient. To determine the required number of motion states in dependence on the motion amplitude, film response distributions were calculated for 2, 3, ..., 30, 31 motion states for each experimental data set.

### Data analysis

#### Film response

The irradiated films were developed (developing machine M35 with developer DX31 and fixer FX31, Kodak GmbH, Stuttgart) and digitized (FIPS Plus LS75, PTW, Freiburg, Germany) in a 1 mm grid as reported by Spielberger et al. [10]. The experimental error of the relative blackening *S*/*S*_0 _was determined in error propagation with ΔS = 0.02 as stated in the manual of the densitometer. Δ*S_0 _*was determined experimentally as described in 2.2. To allow comparison between measured and calculated film responses, each image pair was aligned according to its cross-shaped 2D coordinate system including translations and rotation.

**Figure 2:**
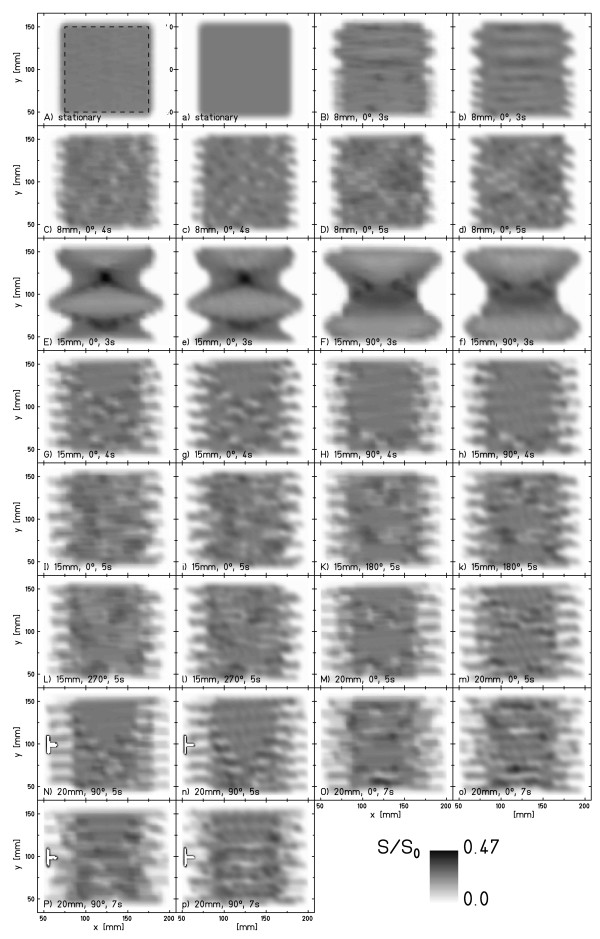
**Results of all parameter combinations**. Measured (A-P) and calculated (a-p) film response distributions for the different parameter combinations (amplitude, initial phase, period). In A) the dashed square indicates the small region-of-interest used in the analysis (ROI_target_).

Quantitative comparisons were performed as follows:

i) For each pixel the difference between experimental and calculated film response was calculated and plotted as [(S/S_0_)_experiment_-(S/S_0_)_calculated_]/(S/S_0_)_experiment_.

ii) Horizontal and vertical line profiles were extracted and plotted for experimental image, calculated image, and difference image including error bars as described above.

iii) The mean difference (± standard deviation, SD) was calculated based on a difference image. This analysis was performed for two regions-of-interest (ROI): The larger ROI, labeled ROI_irradiated_, was defined by all pixels with a calculated film response (S/S_0_)_calculated _> 0.1; the smaller ROI, labeled ROI_target_, included the area of the stationary irradiation only (dashed line in Figure 2a for the pure translational motion).

#### Determination of the number of motion states

For each experimental data set without absorbers (Figure 1a), film response distributions were calculated using 2-31 motion states, i.e. 3D "time-frames" in the 4DCT. The required number of motion states was analyzed qualitatively by visual inspection of the resulting saturation curve. To determine the required number of motion states for a given amplitude quantitatively, the data were fitted with a standard saturation function: *d*(*p*) = *a *· exp(-*b *· *p*) + *c *with *d *relative film response difference and *p *number of motion states. The fitting parameters *a, b*, and *c *represent the initial difference, the saturation rate, and the saturation level of the film response difference at a quasi-continuous number of motion states, respectively. The required number of motion states per amplitude *p_r _*was defined at 5% deviation from saturation level *c*. The required number of motion states *p_r _*was calculated for each measurement. Mean *p_r _*values per motion amplitude as well as individual *p_r _*values with error bars that result from error propagation using the fitting uncertainties of *a, b*, and *c *are reported.

## Results

### Film response calibration

For the experiments without absorbers, analysis of the film response in dependence on the deposited dose resulted in a slope *m *of the blackening curve of *m*_C-12, *E *= 272 MeV/u _= 0.33/Gy with a fitting error of Δ*m *= 0.0125/Gy. The film response in saturation *S*_0 _was 4.0 with a fitting error of Δ*S_0 _*= 0.04. These values were used in all subsequent film response calculations.

### Lateral target motion

#### Stationary reference irradiation

**Figure 3:**
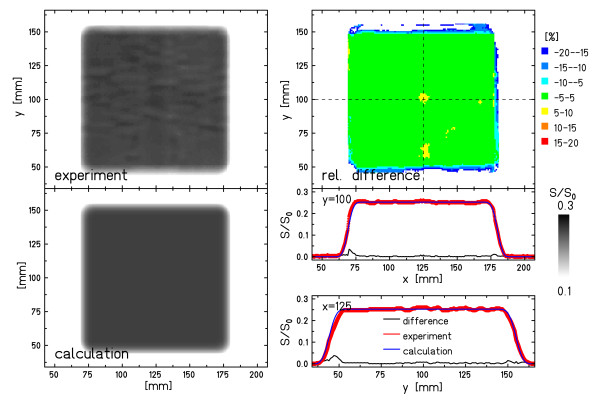
**Film response for stationary irradiation**. Results for the stationary film that were used as reference. The image shows the 2D experimental film response distribution, the calculated distribution, the relative difference between experimental and calculated film response, as well as profiles along the dashed lines in the difference distribution.

For assessment of the film response calculation accuracy for lateral target motion, the setup displayed in Figure 1a was used. Figure 3 shows the results for irradiating a stationary film as a reference for irradiations with target motion. The subfigures show the measured and the calculated film responses, the difference image between calculation and measurement, as well as a horizontal and a vertical line profile. The differences between calculated and measured film responses are -1.52% ± 2.06% inside the ROI_target _and 2.3% ± 4.92% inside the ROI_irradiated_. The largest deviations are observed at the lateral borders in the dose gradients. The profiles demonstrate that these effects are not due to mispositioning.

#### Overview of experiments with lateral motion

Fourteen motion parameter combinations were studied. Amplitudes ranged from 8-20 mm, periods from 3-7 s, and initial motion starting phases from 0-270°. Figure 2 displays experimental as well as calculated film responses (interplay patterns) for the studied motion parameter combinations. Visual inspection yields good agreement between calculation and experiment. Details of the statistical analysis within the two ROIs are provided in Table 1.

**Table 1:** Deviation analysis - single film, lateral motion

				ROI_irradiated_		ROI_target_	
**Figure 2**	**A [mm]**	**φ [°]**	**T [s]**	**Mean [%]**	**SD**	**Mean [%]**	**SD**

a)	0			-2.30	4.92	-1.52	2.06

b)	8	0	3	-2.98	16.92	-0.07	6.31

c)	8	0	4	-2.83	15.56	-0.98	9.16

d)	8	0	5	-2.24	18.53	0.73	9.34

e)	15	0	3	-1.85	10.78	0.66	6.19

f)	15	90	3	-0.89	11.53	0.73	9.50

g)	15	0	4	-3.50	12.06	-0.36	6.74

h)	15	90	4	-4.39	11.19	-1.23	5.17

i)	15	0	5	-5.40	14.99	-1.45	7.75

k)	15	180	5	-5.74	12.74	-2.50	7.61

l)	15	270	5	-6.46	16.09	-2.03	6.35

m)	20	0	5	-7.32	21.52	-2.92	14.49

n)	20	90	5	-5.03	20.64	-0.13	13.62

o)	20	0	7	-4.80	21.76	-0.34	15.08

p)	20	90	7	-9.84	68.98	-2.94	17.02

Mean				-4.52	19.52	-0.92	9.60

SD				2.39	14.74	1.30	3.86

Minimum				-9.84	10.78	-2.94	5.17

Maximum				-0.89	68.98	0.73	17.02

Statistical analysis of relative deviations between calculated and experimental film response in two regions-of-interest (ROI). The film response distributions are shown in Figure 2 for the 14 different motion parameter combinations with amplitude A, period T, and phase φ at start of irradiation.

#### Required number of motion states

**Figure 4:**
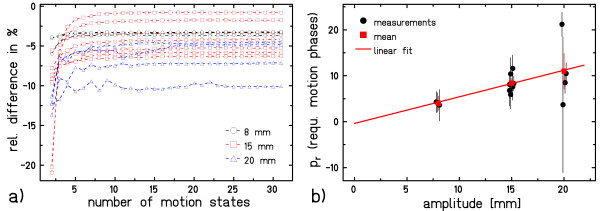
**Influence of the number of motion states**. **a**) Influence of the number of motion states on the relative difference between calculated and experimental film response distribution. Data are grouped according to the motion amplitude. The dashed lines are added to guide the eye. **b**) The required number of motion states p_r _was determined for each measured parameter combination at 8, 15, and 20 mm motion amplitude a. A linear fit based on the mean of each measured motion amplitude yielded *p_r _*= 0.58 · *a *- 0.05.

The dependence of the mean relative difference between measurement and calculation inside the ROI_irradiated _on the number of motion states used for calculations is plotted in Figure 4a grouped for the amplitudes of 8, 15, and 20 mm. All curves plateau above film response calculations with 10-15 motion states. Figure 4b shows the result of the *p_r _*calculation, i.e. the required number of motion states to reach 95% of the plateau value. Despite the comparably large fluctuations at 20 mm amplitude, there is a linear relationship between amplitude *a *and the required number of motion states: *p_r _*= 0.58 · *a *- 0.05. For each 10 mm motion amplitude, ~6 motion states are thus sufficient for dose calculations. If the desired level of agreement is increased to 98% (99%) of the saturation value, the required number of motion states per 10 mm target motion increases to 7 (8).

#### Validation of calculated film response

To exclude influences of the number of motion states *p_r _*on the presented results, calculations for the following analyses were performed with 20 motion states. The results of difference image analysis (mean relative difference) in the two ROIs are given in Table 1 for all 14 image pairs. Agreement between experimental and calculated film response is higher in the smaller ROI_target _(-0.92% ± 1.30%) than in the larger ROI_irradiated _(-4.52% ± 2.39%). In general, agreement is lower for the four distributions at 20 mm motion amplitude.

**Figure 5:**
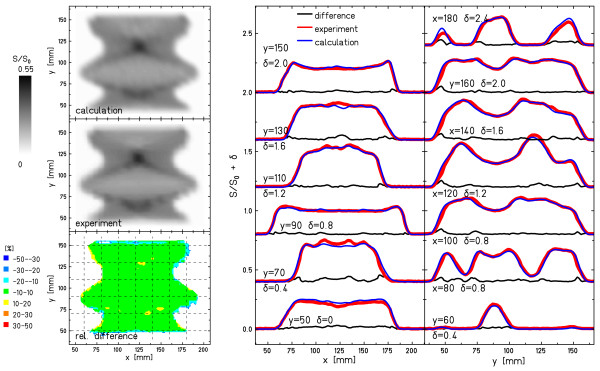
**Specific example: good agreement**. Example of good agreement between experimental and calculated film response (Figure 2e). Apart from the distributions of the experimental response, the calculated response, and the relative difference, horizontal and vertical profiles are shown along the dashed lines indicated in the distribution of the relative difference. Details of the analysis are reported in Table 1. δ indicates the vertical shift of the line profiles in order to show all of them in the same figure.

**Figure 6:**
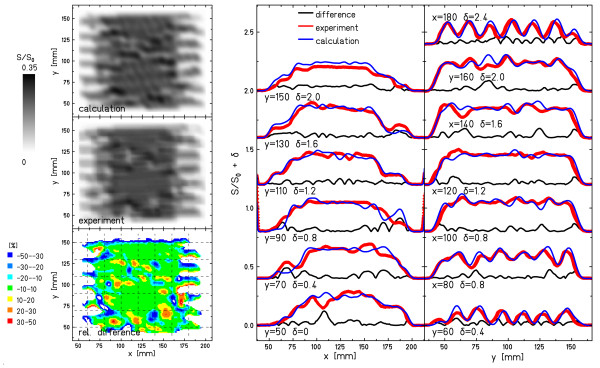
**Specific example: less good agreement**. Example of less good agreement between experimental and calculated film response (Figure 2o). Apart from the distributions of the experimental response, the calculated response, and the relative difference, horizontal and vertical profiles are shown along the dashed lines indicated in the distribution of the relative difference. Details of the analysis are reported in Table 1. δ indicates the vertical shift of the line profiles in order to show all of them in the same figure.

Figures 5 and 6 show examples for a motion parameter combination with good agreement between measurement and calculation in the statistical analysis (Figure 5) and a parameter combination with lower agreement (Figure 6). Both images show the experimental and calculated film response distributions, the relative differences to calculations, as well as horizontal and vertical line profiles.

For both examples, the line profiles indicate that the calculation reproduced the measured interplay pattern well. While in Figure 5 the deviations are negligible, the example with lower agreement in Figure 6 reveals deviations mainly at the transitions from irradiated to unirradiated areas and in the fine-structure of the interplay pattern (e.g. the "cross" centered at x = 125 mm/y = 125 mm or the area 50 mm < y < 75 mm).

### Lateral target motion including range changes

For assessment of combined lateral and longitudinal target motion, four different experiments each with a stack of 5 films were performed in comparison to a stationary experiment with the same setup. Motion parameters of the individual experiments are listed in Table 2.

**Table 2:** Motion parameters of film stack experiments

Experiment	no2	no3	no4	no5
Period [s]	4.7	5.9	5.9	2.9

φ [°]	174	182	346	202

Motion parameters (period and φ phase at start of irradiation) of the filmstack experiments to assess lateral target motion including range changes. All films were moving at an amplitude of 19 mm.

**Figure 7:**
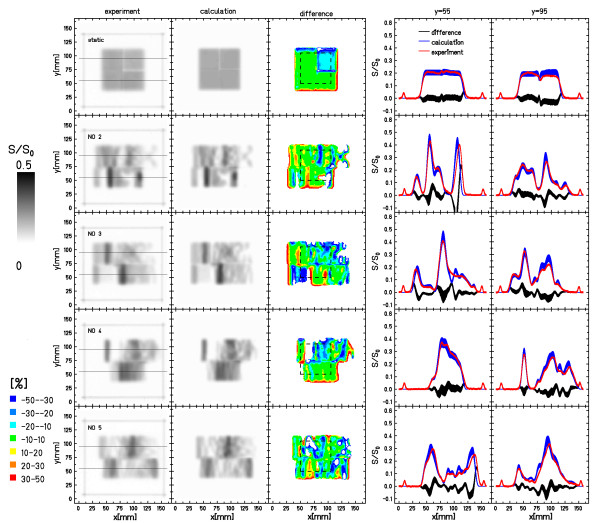
**Film stack measurements - all parameter combinations**. Results of the film stack measurements that included ranges. Displayed are the data for film 3 for the stationary irradiation as well as the 4 irradiations that included lateral motion. The distributions show experimental response, calculated response, their relative difference, as well as profiles along the horizontal lines indicated in the experimental distributions. Details of the analysis are reported in Table 3.

**Figure 8:**
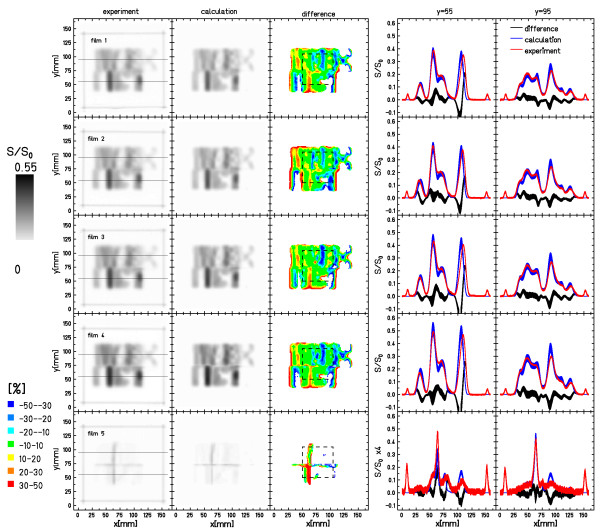
**Film stack measurements - experiment NO2**. Results of the film stack measurements that included ranges. Displayed are the data of all films for experiment NO2 (see Table 3 and Figure 7). The distributions show experimental response, calculated response, their relative difference, as well as profiles along the horizontal lines indicated in the experimental distributions. Details of the analysis are reported in Table 3. Note that the profiles for film 5 are scaled by a factor of 4.

Figures 7 and 8 show the results of these measurements that were performed with the setup shown in Figure 1b. Figure 7 shows the results for film 3 of all measurements (stationary reference and four different motion parameter combinations). The direct comparison of calculated and experimental film response distributions shows good agreement. For the right-top part of the pattern (bolus: 20 mm, see Figure 1c), there are deviations of > 10% between experimental data and calculation even for irradiation of a stationary film (setup in Figure 1a). For experiments with target motion, the calculations reproduced the distinct interplay patterns. Comparison of the two horizontal profiles yields similar results but the profiles also reveal that positions of some interplay-induced peaks slightly deviate for some of the experiments (e.g. NO2 and NO5, y = 55 mm).

In Figure 8, the responses of all 5 films in the stack for measurement NO2 of Figure 7 are shown. The calculations reproduce the experimental film responses well. Interference patterns as well as height of the peaks that is dominated by the depth of the films in the stack are modeled within the experimental errors with the exemption of some peak positions.

Analysis of the difference images shows that the deviations between experiments and calculations are within 10% for most of the irradiated area (green regions in Figure 8). Comparable to the stationary setup, the top-right part of the irradiations with target motion indicates slightly higher deviations. The differences in some peak positions results in blue or red bands in the difference images that are also prominent at some transition areas from irradiated to unirradiated regions as for the results for lateral motion only (section 3.2).

Table 3 shows the statistical data of the pixel-by-pixel comparison between measured and calculated data. The mean relative deviation in ROI_irradiated _is -11.1% (SD 11.7%) for ROI_target _the deviations are -10.3% (SD 9%) in comparison to -13.1% (SD 20.9%) and -13.5% (SD 19.9%) for the stationary irradiation. If film #5 that is positioned in the distal fall-off region is omitted from the analysis the values change to -6.0% (SD 2.5%)/-6.4% (SD 2.6%) in comparison to -2.7% (SD 3.3%)/-3.6% (SD 2.5%) for the stationary irradiation for ROI_irradiated_/ROI_target_.

**Table 3:** Deviation analysis - film stack

ROI_irradiated_													
	**Film**					**all films**				**without film 5**			

**experiment**	**#1**	**#2**	**#3**	**#4**	**#5**	**mean**	**SD**	**min**	**max**	**mean**	**SD**	**min**	**max**

stationary	-4.5	--	-4.8	1.1	-44.3	-13.1	20.9	-44.3	1.1	-2.7	3.3	-4.8	1.1

no2	-4.2	-2.8	-3.2	-7.1	-11.2	-5.7	3.5	-11.2	-2.8	-4.3	1.9	-7.1	-2.8

no3	-10.3	-5.5	-10.6	-2.5	-43.1	-14.4	16.4	-43.1	-2.5	-7.2	3.9	-10.6	-2.5

no4	-3.6	-2.9	-6.2	-2.8	-36.8	-10.5	14.8	-36.8	-2.8	-3.9	1.6	-6.2	-2.8

no5	-6.9	-7.3	-12.7	-8.0	-35.0	-14.0	12.0	-35.0	-6.9	-8.7	2.7	-12.7	-6.9

mean	-6.2	-4.6	-8.2	-5.1	-31.5	-11.1	11.7	-31.5	-3.7	-6.0	2.5	-9.2	-3.7

SD	3.0	2.2	4.3	2.8	14.0								

min	-10.3	-7.3	-12.7	-8.0	-43.1								

max	-3.6	-2.8	-3.2	-2.5	-11.2								

ROI_target_													

	Film					all films				without film 5			

experiment	#1	#2	#3	#4	#5	mean	SD	min	max	mean	SD	min	max

stationary	-1.3	-	-6.3	-3.3	-43.2	-13.5	19.9	-43.2	-1.3	-3.6	2.5	-6.3	-1.3

no2	-4.7	-3.3	-5.2	-7.5	-14.0	-6.9	4.2	-14.0	-3.3	-5.2	1.8	-7.5	-3.3

no3	-6.4	-3.5	-8.9	-4.2	-33.5	-11.3	12.6	-33.5	-3.5	-5.7	2.4	-8.9	-3.5

no4	-0.6	-3.2	-9.6	-3.3	-21.5	-7.7	8.4	-21.5	-0.6	-4.2	3.8	-9.6	-0.6

no5	-8.3	-9.0	-13.8	-11.5	-33.9	-15.3	10.6	-33.9	-8.3	-10.7	2.5	-13.8	-8.3

mean	-5.0	-4.8	-9.4	-6.6	-25.7	-10.3	9.0	-25.7	-3.9	-6.4	2.6	-10.0	-3.9

SD	3.3	2.9	3.5	3.7	9.7								

min	-8.3	-9.0	-13.8	-11.5	-33.9								

max	-0.6	-3.2	-5.2	-3.3	-14.0								

Mean deviation (in %) between calculated and measured film response *S*/*S*_0 _determined in the two ROIs for the experimental setup with combined lateral and longitudinal motion. "all films" and "without film 5" show the data for a certain experiment. The data underneath the outcome for the individual films reflect the outcome per films in the 4 experiments with target motion. Film #2 (stationary) was damaged in the development process.

## Discussion

Experiments with moving radiographic films were performed with the intention to validate the calculations of our 4D treatment planning system in the presence of translational target motion. For non-rigid motion patterns the calculation routines will be identical but non-rigid registration routines need to be incorporated and validated which was not part of this study. Calculation routines are in principle identical for rescanning [4], beam gating [7,17,18], and also beam tracking [8,9,19]. Since the calculation of film responses relates on the calculation of the delivered dose, the presented results therefore form the basis of 4D dose calculation precision for motion mitigated irradiation schemes. A full treatment plan validation needs to incorporate optimization parameters that depend on the motion mitigation technique and should be complemented with, e.g., ionization chamber data that have a higher accuracy than radiographic films at a lower spatial resolution. The validation of 4D optimization parameters such as compensation vectors for beam tracking, the size of the gating window in gated irradiations, or the number of required rescans as well as aspects of biological treatment planning have not been investigated.

Irradiations of moving detectors without motion mitigation were chosen for experimental validation because resulting dose distributions provide prominent interplay patterns and are very sensitive to slight parameter changes and thus also to slight mismatches in the calculation. To measure interplay patterns accurately, a detector with high spatial resolution is required. Radiographic films are ideally suited for this purpose despite their dependence on the irradiation field and their limited accuracy. For mixed irradiation fields that are, e.g., given in our experimental setup (Figure 1b) for combined lateral and longitudinal motion, analysis of the data reported by Spielberger et al. yields an agreement between measurement and calculation of ~10% in the target regions and within 30% at the border of the irradiation field for stationary irradiations [10]. To exclude the dependency on the composition of the irradiation field, we also performed experiments in which the films were irradiated with a single particle energy in the plateau region of the Bragg peak (setup shown in Figure 1a). This irradiation scheme allows a calibration of the detector as described in section 2.2.1., which reduced the uncertainty at least in the central parts of the irradiation field (stationary irradiation in Figure 3). For the borders, i.e. the transitions from irradiated to unirradiated areas, the precision of the detector is mainly determined by the beam shape. Imperfections such as deviations from the nominal Gaussian beam shape lead to deviations that were visible as the blue colored "margin" (rel. difference > 10%) in Figure 3, 5, and 6. In the central part of the target volume these deviations from the nominal beam profile lead to stripe patterns in the case of stationary beam delivery (Figure 3) with an acceptable dosimetric impact because neighboring beam spots have a sufficiently large overlap. In case of a moving detector, interplay results in a decreased overlap and the dose contribution of a single beam position has a higher impact. In the experiments with the mixed irradiation field, the necessity of a precise beam shape is in general less prominent since there is additional dose contribution from neighboring iso-energy slices. However, also Spielberger et al. reported increased deviations of up to 30% at the borders of the irradiation field [10]. Due to these slight beam shape distortions but also due to the uncertainties in the extraction rate and motion trajectory measurements the deviations in calculated film response distributions in the presence of motion are expected to be higher than for irradiations of stationary detectors.

With increasing motion amplitude interplay effects increase and therefore areas with transitions from irradiated to unirradiated increase. This explains the larger deviations between measured and calculated film response distributions for 20 mm motion amplitude. The effect is also visible by comparing the results of the lateral target motion for the large ROI that includes the complete film response distribution and the small ROI which encompasses only the central region of the films. Agreement is better within the small ROI: maximum mean deviation -2.94% and a maximum SD of 17.02% in comparison to a maximum mean deviation of 9.84% (SD 68.98%) in the large ROI. For the lateral motion including range changes the deviations between the two ROIs is less pronounced.

A comparison between experiments in the plateau region of the Bragg peak with only lateral motion and the film stack experiments in the Bragg peak region that included range changes shows an increased deviation between calculation and experiment for the film stack measurements. This is most likely due to the additional sensitivity of the calculations to the exact description of the experimental setup. Already in the stationary experiment, the deviations are increased especially for film #5 that is positioned in the distal fall-off region of the Bragg peak which is most sensitive to position deviations due to the sharp gradient. Similar arguments are valid for films 1-4 since we did not irradiate a spread-out Bragg peak but a pristine peak. The most likely reasons for position deviations are uncertainties in the bolus thicknesses and the range precision in the treatment planning process.

The determined number of 6 required motion states per 10 mm motion amplitude is only valid for the distal slice of an extended target volume. In more proximal regions intrinsic averaging effects, namely the dose contributions from irradiation of more distal slices in proximal slices, lead to reduced sensitivity to target motion [18]. If 6 states per 10 mm motion amplitude are sufficient for 4D calculation as well as optimization, standard 4DCT protocols with ~10 reconstructed motion states will be sufficient for typical intra-fractional motion amplitudes of, e.g., lung tumors [20,21].

## Conclusion

GSI's 4D treatment planning system TRiP [1,2] was validated by comparing calculated film responses to experimental data for translational motion geometries. Experimental and calculated film responses for lateral motion without range changes show good agreement of -0.92 ± 1.3% in an region of interest covering the target area in comparison to < 10% reported by [10] for stationary targets. If range changes are introduced, the agreement is still given with a deviation of -6.4 ± 2.6% (-10.3 ± 9.0% if film #5 in distal fall-off is included) in comparison to -3.6% (-13.5% including film #5) for the stationary irradiation. By performing calculations with varying number of motion states 6 motion states per 10 mm motion amplitude were determined to be sufficient.

## Competing interests

GSI Biophysics currently receives funding from Siemens AG, Healthcare Sector, Imaging & Therapy, Particle Therapy. ER currently is an employee of Siemens AG, but most of the work in the presented manuscript was performed prior he entered Siemens AG.

## Authors' contributions

CB, ER: design of study, CB data acquisition and majority of data analysis, all: data analysis and manuscript generation. All authors read and approved the final manuscript.
